# The Diagnostic Efficiency of Ultrasound Computer–Aided Diagnosis in Differentiating Thyroid Nodules: A Systematic Review and Narrative Synthesis

**DOI:** 10.3390/cancers11111759

**Published:** 2019-11-08

**Authors:** Nonhlanhla Chambara, Michael Ying

**Affiliations:** Department of Health Technology and Informatics, The Hong Kong Polytechnic University, Hung Hom, Kowloon, Hong Kong, SAR, China; nonhlanhla.chambara@connect.polyu.hk

**Keywords:** computer-aided diagnosis, thyroid nodules, grey scale and Doppler ultrasound, Thyroid Imaging Reporting and Data System

## Abstract

Computer-aided diagnosis (CAD) techniques have emerged to complement qualitative assessment in the diagnosis of benign and malignant thyroid nodules. The aim of this review was to summarize the current evidence on the diagnostic performance of various ultrasound CAD in characterizing thyroid nodules. PUBMED, EMBASE and Cochrane databases were searched for studies published until August 2019. The Quality Assessment of Studies of Diagnostic Accuracy included in Systematic Review 2 (QUADAS-2) tool was used to assess the methodological quality of the studies. Reported diagnostic performance data were analyzed and discussed. Fourteen studies with 2232 patients and 2675 thyroid nodules met the inclusion criteria. The study quality based on QUADAS-2 assessment was moderate. At best performance, grey scale CAD had a sensitivity of 96.7% while Doppler CAD was 90%. Combined techniques of qualitative grey scale features and Doppler CAD assessment resulted in overall increased sensitivity (92%) and optimal specificity (85.1%). The experience of the CAD user, nodule size and the thyroid malignancy risk stratification system used for interpretation were the main potential factors affecting diagnostic performance outcomes. The diagnostic performance of CAD of thyroid ultrasound is comparable to that of qualitative visual assessment; however, combined techniques have the potential for better optimized diagnostic accuracy.

## 1. Introduction

Thyroid nodules are a common finding in symptomatic and asymptomatic patients and have a malignancy risk rate of about 5–15% [1]. However, the incidence of thyroid cancer is rising due to the increased sensitivity in diagnostic imaging tools such as ultrasound [2,3]. Fine-needle aspiration cytology (FNAC) is the reference standard preoperatively; however, it is minimally invasive and can yield non-diagnostic results in about 25% of the samples and about 20–30% indeterminate results [4]. Current thyroid management guidelines recommend ultrasound for the primary investigation of all suspected thyroid nodules and FNAC being reserved for further investigation of suspicious or equivocal ultrasound findings [5,6]. The primary goal in the diagnosis of thyroid nodules is to limit unnecessary FNAC procedures and unwarranted thyroid surgery on benign nodules which may lead to cost and quality of life implications.

Ultrasound is an operator-dependent imaging modality whose results are prone to subjective interpretation. Subjective assessment in grey scale thyroid ultrasound is dependent on the presence of features suggestive of malignancy or benignity; with multiple features within a single nodule having higher predictive value and diagnostic accuracy [7,8]. Thyroid malignancy risk stratification guidelines such as the thyroid imaging reporting and data system (TIRADS) are used in routine clinical practice to differentiate benign and malignant nodules. Some of the guidelines used include those from the American Thyroid Association (ATA), American College of Radiology (ACR), Korean Society of Thyroid Radiology (KSThR), European Thyroid Association (EU), and the American Association of Clinical Endocrinologists, the American College of Endocrinology, and the Associazione Medici Endocrinologi (AACE/ACE/AME) [5,6,9,10,11,12].

Computer-aided diagnosis (CAD) systems have emerged in past years as non-invasive approaches to complement radiologists’ interpretation and potentially overcome subjective interpretation limitations. CAD detection and diagnosis methods are based on machine learning approaches that use statistical and data mining algorithms, which rely on textural ultrasound features and quantitative regional segmentation of vascularity, to differentiate benign and malignant nodules [13,14,15,16]. CAD software can be embedded within the ultrasound unit or be used as an isolated program for offline image analysis. Grey scale ultrasound CAD software is equipped with selected TIRADS for diagnostic purposes, whereas Doppler ultrasound CAD is often based on stipulated cut-off points for vascularity indices (VI) used in differentiating peripheral and central vascularity in thyroid nodules.

Presently, few studies have investigated the diagnostic performance of various thyroid ultrasound CAD methods as applied in the clinical context and shown variable results. Existing systematic reviews have been based on analyzing different textural techniques and machine learning algorithms and classifiers more on a biomedical engineering perspective rather than clinical applicability [13,17,18,19]. A recent systematic review and meta-analysis study focused on the diagnostic performance of mainly the Samsung S-Detect CAD software in comparison with that of radiologists for the differentiation of thyroid nodules in clinical settings [20]. As various CAD systems for ultrasound feature assessment of thyroid nodules have been developed by various researchers and clinicians in different parts of the world, there are multiple factors that can influence their diagnostic performance.

To the best of our knowledge, there is currently a lack of reviews analyzing the performance of different types of ultrasound CAD for characterizing thyroid nodules. Hence, this present systematic review assesses and summarizes current evidence on the diagnostic performance of various thyroid ultrasound CAD software in the differentiation of benign and malignant thyroid nodules and identifies potential factors that may influence diagnostic efficiency and clinical applicability.

## 2. Materials and Methods

### 2.1. Literature Search

The following electronic databases were searched: PUBMED, EMBASE and Cochrane Library. The search strategy was based on the PICOS framework to search concepts relating to the population, intervention, and outcomes in the different databases. The search concepts were: (1) Thyroid neoplasm, (2) ultrasonography, (3) computer-assisted diagnosis, and (4) diagnostic accuracy, and their related terms as MeSH terms, keywords and/or EmTree terms. Initially, there were no date or language restrictions. Searches were re-run regularly until August 2019 before the final analyses to retrieve more studies for inclusion.

### 2.2. Inclusion and Exclusion Criteria

All the studies analyzed for assessing the diagnostic performance of thyroid ultrasound computer-aided diagnosis techniques had to meet the following criteria: (1) The study involved only human subjects and had institutional ethical approval; (2) informed consent was either obtained from each participant or adequately waived for retrospective studies; (3) the study investigated the diagnostic performance of computer-aided diagnosis techniques in either grey scale or Doppler ultrasound or both for differentiation of benign and malignant thyroid nodules in a clinical setting; (4) use of an appropriate reference standard (FNAC or histopathology); (5) diagnostic performance outcomes of interest were reported in terms of sensitivity, specificity, negative predictive values (NPV), positive predictive values (PPV), diagnostic accuracy, and/or area under receiver operator characteristic curve ROC curve (AUROC); and (6) peer-reviewed articles in English. Exclusion criteria were: (1) Unrelated to computer-aided diagnosis in ultrasound of thyroid nodules; (2) reviews, case reports, editorial letters, or commentaries; (3) studies assessing engineering classifiers of thyroid ultrasound textural features; (4) non-English; and (5) insufficient diagnostic accuracy outcomes.

### 2.3. Data Extraction

Two reviewers independently performed data extraction and discrepancies were resolved by reaching a consensus. A standardized data abstraction form was developed based on the preferred reporting items for systematic reviews and meta-analysis (PRISMA) guidelines [21,22]. For each study included in this review, the following information was extracted: Authors, year of publication, number of patients and thyroid nodules, average thyroid nodule diameter, the reference standard for final diagnosis, type of ultrasound machine and transducer used, type of computer-aided diagnosis used, and optimal cut-off points for determining malignancy where applicable. The specificity, sensitivity, NPV, PPV, and diagnostic accuracy or AUC from each study were extracted from each reported study.

### 2.4. Quality Assessment

The risk of bias and methodological quality of the included studies was assessed using the QUADAS-2 checklist tool [23]. The included studies were assessed on the four major quality domains: (1) Patient selection bias and applicability; (2) index test conduct and interpretation bias and applicability; (3) reference standard, its conduct and interpretation bias and applicability; and (4) patient flow and timing bias and applicability. Each of these domains was categorized as high, low, or unclear and discrepancies resolved by consensus upon re-reviewing the articles.

### 2.5. Data Analysis Approach

Due to the wide range of the diagnostic criteria in the studies included in this review, a meta-analysis was not done as few studies had comparable criteria. A narrative synthesis was adopted, and the analysis focused on categorizing findings based on the type of ultrasound features, comparative analysis of human and CAD performance, as well as the TIRADS used.

## 3. Results

### 3.1. Literature Search

**Figure 1:**
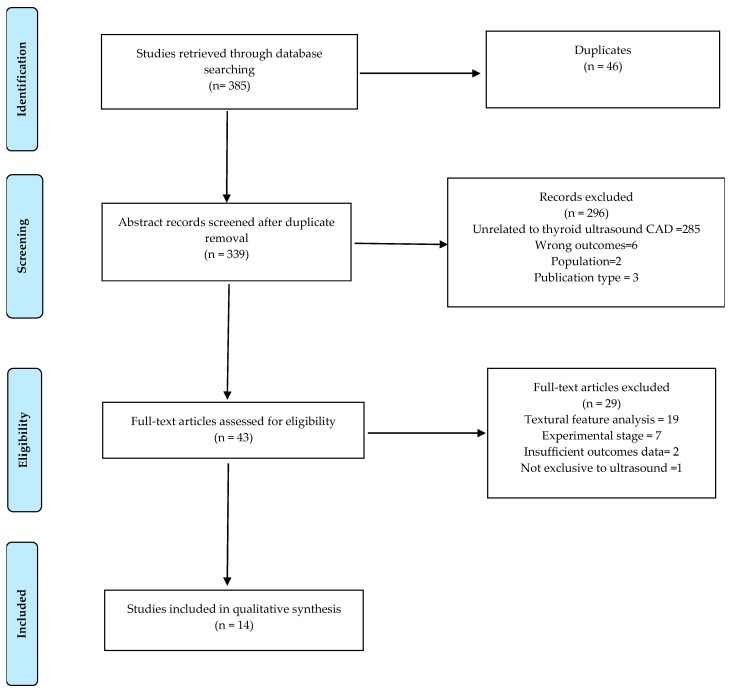
Flow chart of the study selection process.

The initial comprehensive search strategy up to 15 August 2019, yielded 385 papers for the title and abstract screening with 46 duplicates being excluded (Figure 1). There were 203 articles from PUBMED, 165 articles from EMBASE and 17 articles from Cochrane. A further 296 papers were excluded based on the abstract review as they were not related to the review topic. Of these, 285 papers were unrelated to thyroid ultrasound computer-aided diagnosis. Six studies were excluded based on wrong outcomes, with two focusing on computer-assisted scintigraphy [24,25] while the other four focused on quantitative elastography [26], computed-tomography [27], laser ablation [28], and sonographic localization of metastatic lymph nodes [29]. Because the scope of this review was limited to human populations with thyroid nodules, two studies were excluded with one being a mouse model study [30] and the other a human cell line study [31]. Two review articles and an editorial letter were excluded as they did not meet the publication inclusion criteria of original research articles [17,19,32]. A full-text review was conducted for the remaining 43 papers which resulted in the exclusion of 29 articles that did not meet the inclusion criteria. Nineteen textural feature analysis studies were excluded because they focused on texture feature extraction and classifier performance for the design and developmental phase of CAD software from an engineering perspective rather than a clinical approach. Of these, seven analyzed the performance of various statistical textural features [33,34,35,36,37,38,39], while six studies analyzed performance of a combination of textural features and other features, namely texture and wavelet transform features [40,41,42], texture and morphological features [43], and texture and radiological features [44], as well as texture analysis, elastography and grey scale ultrasound [45]. Two studies evaluated the performance of the combination of histogram and fractal texture analysis for support vector machine (SVM) and random forest classifiers [46,47] and one study assessed the accuracy of wavelet texture analysis for different classifiers [48]. Three studies focused on artificial intelligence texture analysis; two evaluated the diagnostic performance of the combination of artificial neural network (ANN) textural analysis with SVM [49], and ANN with binary logistic regression analysis [50], while another evaluated deep learning convolutional neural network feature classification performance using a random forest classifier [51]. Seven studies were excluded because they were pre-clinical pilot studies for the validation of different CAD algorithms and classifiers [52,53,54,55,56,57,58]. Two studies were excluded based on insufficient diagnostic performance outcomes as one had insufficient data to determine sensitivity, specificity, and diagnostic accuracy for adequate comparative analysis between the CAD software and radiologist [59,60] while one study was not exclusively on ultrasound [61]. A total of 14 papers met the inclusion criteria for this review.

### 3.2. Study Characteristics

All studies included in this review were diagnostic cohort studies comprising of eight prospective studies [62,63,64,65,66,67,68,69] and six retrospective studies [70,71,72,73,74,75] published between 2007 and 2019. The main patient characteristics in these studies are summarized in Table 1. The total number of patients from the 14 studies was 2232. The number of patients ranged from 50 to 333 patients per study with an average female percentage of 79% ± 4.3 (range 69.7–85%) across the included studies although two studies did not give gender distribution [26,32]. From these patients, a total of 2675 thyroid nodules (1396 benign and 1279 malignant) were evaluated and the mean malignancy rate was about 46% ± 16.9 (22.6–78.8% range). In the studies that reported the mean diameter of the thyroid nodules, the range was from 1.5 to 3.37 cm for benign nodules and 0.9 to 3.2 cm for malignant nodules. The reference standard, type of ultrasound machine, and CAD characteristics are outlined in Table 2. Four studies used the Philips HDI 5000 ultrasound system (Philips Healthcare, Bothell, WA, USA) [62,63,64,72], four studies used the Samsung RS80A (Samsung Medison, Gyeonggi-do, Republic of Korea) [65,67,68,71], while three studies included multiple ultrasound systems [70,73,75], and another three used Aixplorer (Supersonic Imagine Aix en Provence, France), Sonoline Elegra (Siemens Healthcare, Erlangen, Germany) and GE Logiq E9 (GE Healthcare, Chicago, IL, USA), respectively [66,69,74].

Five studies used histopathology exclusively as a reference standard [69,70,72,74,75] while one used FNAC only [71], and eight studies used both histopathology and FNAC [62,63,64,65,66,67,68,73]. Ten studies assessed the diagnostic performance of CAD of grey scale ultrasound features with two focusing solely on calcifications while one focused on echogenicity, and four studies assessed Doppler vascularity features. Four studies used S-Detect for thyroid CAD (Samsung Medison, Gyeonggi-do, Republic of Korea) embedded in Samsung ultrasound systems [65,67,68,71] whilst another four used AmCAD-UT CAD software (AmCad BioMed, Taipei, Taiwan) [62,63,64,74]. Three studies used self-developed artificial intelligence (AI) CAD systems [70,72,75]. The remaining three studies employed self-developed Doppler ultrasound CAD algorithms [66,69,73]. The diagnostic performance outcomes of the different thyroid ultrasound CAD systems in the included studies are summarized in Table 3.

### 3.3. Quality Assessment

The quality assessment of the included studies for bias and applicability using the QUADAS-2 tool is summarized in Table 4 and Table 5, respectively. Most studies avoided case-control design, had appropriate reasons for exclusions, and blinded the reference standard to index test. All studies had a reference standard; however, six studies had a high risk of patient selection bias due to the recruitment of patients scheduled for thyroid surgery and retrospective analysis of ultrasound features for the determination of the diagnostic performance of thyroid ultrasound CAD. Applicability concerns in reference to patient selection, index test, and reference standard definition of the condition were low risk in most of the studies. The PRISMA-2009 checklist (Table S1) and graphical display of the risk of bias (Figure S1) and applicability concerns (Figure S2) are provided in the Supplementary Materials.

### 3.4. Study Findings

This section covers the narrative synthesis and summaries of study findings. The synthesis of the findings was guided by the different types of CAD studies included in this review. To ensure the logical comparative analysis of the diagnostic performance outcomes, the different studies were categorized to create subsections based on the relatedness of ultrasound features and the similarities in the study methodological approaches. The subsections begin with studies that focused on the performance of CAD of isolated grey scale ultrasound features, followed by CAD performance of Doppler ultrasound features, and lastly a subsection on general CAD performance which is further subdivided into an analysis of diagnostic performance between CAD and radiologists and diagnostic performance under different TIRADS guidelines.

### 3.5. Performance of Sole Computerised Ultrasound Features

#### 3.5.1. Echogenicity

One study in the review evaluated CAD diagnostic performance in evaluating echogenicity. Wu et al. [63] compared the diagnostic performance of human assessment of echogenicity and computed quantified analysis of echogenicity based on echogenicity indices for hypoechogenicity and marked hypoechogenicity based on computed mean grey value differences between the nodule and normal thyroid tissue (EI_N–T_), and the nodule and strap muscle (EI_N–M_ less), respectively. Their results showed that marked hypoechogenicity based on the computed quantitative echogenicity index was very specific (93.3%) but had a low sensitivity (33.1%) as compared to the visual human assessment of hypoechogenicity which was highly sensitive (89.8%) but low on specificity (31.9%). Although the study found computed hypoechogenicity to be independently predictive and highly specific for thyroid malignancy, the authors indicated the need to combine it with other computed ultrasound features to improve diagnostic performance. Further studies are necessary to validate these findings and to assess the diagnostic performance of combined CAD methods.

#### 3.5.2. Echogenic Foci

Two studies in this review assessed ultrasound CAD of thyroid nodule calcifications based on computed calcification at different threshold settings and showed varying results. Chen et al. [64] compared the diagnostic performance of computerized quantitative analysis of ultrasound calcifications and human assessment by experienced sonographers and their results showed higher sensitivity with CAD (80%) than qualitative assessment (48.2%) but with a lower specificity for CAD (55%) and a higher specificity for qualitative assessment (89%). The authors highlighted that using the quantified CI, the choice of threshold can be adjusted to prioritize higher sensitivity over specificity to rule out calcium-filled cancers. Choi et al. [72] assessed the diagnostic accuracy of computed calcification analysis using a neural network in differentiating benign and malignant thyroid nodules and found the diagnostic performance to be optimal for both sensitivity and specificity (83% and 82.4%, respectively). The study indicated that quantified interpretation of thyroid nodule calcifications may improve efficiency and consistency in thyroid nodule diagnosis. Due to the limited number of studies evaluating CAD of thyroid nodule calcifications, and with diverse methodological approaches in the two studies in this review, conclusive inferences cannot be adequately drawn, hence more studies are warranted to assess the diagnostic performance of CAD for this ultrasound feature.

#### 3.5.3. Doppler Ultrasound Feature

Four studies evaluated the diagnostic performance of CAD of Doppler ultrasound features. Wu et al. [62] assessed the diagnostic performance of computed thyroid power Doppler vascular indices (VI) for central and ring vascularity densities at different cut-off points in determining thyroid malignancy in power Doppler images. Results from the study showed that using the minimum value (PDVI_min_) of the central VI at a threshold of 5.453 as diagnostic criteria, specificity was higher (89.3%), with a sensitivity of 40.5% and an accuracy of 73.1%. The sensitivity was higher (84.8%) and the specificity lower (40.9%) when the average VI at a threshold of 37.056 was used as a screening tool. However, the authors indicated that benign nodules predominantly had more intranodular vascularity and higher vascularity VIs in both central and ring regions than malignant nodules in this study. Intranodular vascularity was therefore not a reliable predictor of malignancy in this study, however, quantified VIs may be useful for differentiation of benign and malignant nodules when acceptable thresholds are chosen for optimized sensitivity and specificity.

Baig et al. [66] compared the diagnostic performance of qualitative grey scale ultrasound feature evaluation, quantitative regional color Doppler vascularization VIs, and the combined VIs with qualitative grey scale ultrasound analysis. In their study, the combination of Doppler CAD VIs of color Doppler ultrasound images with the qualitative assessment of grey scale ultrasound features resulted in improved specificity from 46.4% to 83.3%, improved diagnostic accuracy from 58.6% to 79.3%, but reduced sensitivity from 96.3% to 66.7% from initial sole qualitative analysis. Combined VIs alone optimized sensitivity (70.4%), specificity (71.4%), and accuracy (71.2%), although accuracy was still slightly lower than that of combined VIs with qualitative assessment. The highest accuracy achieved with the combination of both quantified vascularity and qualitative assessment in this study suggests the potential for improved diagnostic performance with combined methods.

Lyshchik et al. [69] compared the diagnostic performance of human qualitative vascularization assessment of power Doppler images with quantitative intranodular vascularization based on normalized and weighted VIs. Their findings showed that qualitative human assessment of increased intranodular vascularity had a low diagnostic performance with a sensitivity of 65.2% and overall accuracy of 58.9% for all thyroid nodules but higher specificity (85.7%) and diagnostic accuracy (72.1%) for thyroid nodules <2 cm. Furthermore, for benign lesions, intranodular vascularization increased with increase in the size of the lesions, with increased intranodular vascularization observed in only 14.3% of lesions <2 cm but 65.4% of those >2 cm. Quantitative analysis in the same study showed that the size of the thyroid nodule influenced the diagnostic performance of the Doppler algorithm for both normalized and weighted VI at stipulated cut-off points. For all thyroid nodules, the VIs were poor discriminators of benign and malignant nodules, however, normalized VI cut-off point yielding 82.5% sensitivity, 54.3% specificity, and 68.4% diagnostic accuracy. However, thyroid nodules <2 cm had a better diagnostic performance with 72.4% sensitivity, 100% specificity, and 86.2% diagnostic accuracy for normalized VI. These findings suggest that size of a thyroid nodule may influence the diagnostic performance of vascular indices, however this evidence is limited as this was the only Doppler study that had a nodule size-based vascularity analysis.

Sultan et al. [73] compared qualitative color Doppler vascularity evaluation and quantitative central vascular area and central flow volume analysis. The study findings demonstrated that quantitative vascularity assessment based on the central vascular area was more sensitive (90%) than qualitative vascular assessment (67.5%), respectively, with a diagnostic accuracy of 89%. These findings affirm that quantifying intranodular vascularity based on automated zonal segmentation is more objective in assessing vascularity to differentiate benign and malignant thyroid nodules. These findings, however, differ from those of Baig et al. [66] who found a lower sensitivity than qualitative analysis of central vascularity. This may be attributed to the differences in methods.

Among all the Doppler ultrasound CAD studies, based on quantitative vascularity analysis, the highest sensitivity observed was 90% whilst the highest specificity was 100%. The setback of the Doppler ultrasound CAD software and algorithms in these studies is that they are not real-time or embedded within the ultrasound machine and often require offline analysis. Furthermore, the studies have different methodologies of assessing vascularity and calculating vascularity indices and optimal cut-off points for differentiating benign and malignant nodules.

### 3.6. General Performance of CAD

#### 3.6.1. Performance between CAD and Radiologists (Clinicians)

Five studies focused on the general performance of grey scale CAD and human visual assessment by radiologists or clinicians based on the same TIRADS guideline for both approaches. Yoo et al. [65] compared the diagnostic performance of an experienced radiologist using the KSThR-TIRADS guidelines [11], sole CAD system, and the radiologist assisted by the CAD system. The results from the study showed that the radiologist visual assessment had a higher specificity (95.5%), while the sensitivity for both CAD and radiologist assessment was comparable. A combination of the radiologist and CAD assessment yielded higher sensitivity (92%) than sole approaches, but with slightly lower specificity (85.1%). The diagnostic accuracy of all three approaches was comparable, with that of the radiologist being slightly higher (90.6%).

Jeong et al. [68] evaluated the diagnostic performance of an experienced radiologist using KSThR TIRADS and the CAD system used by four operators with different levels of experience in ultrasound, ranging from 0–10 years including the experienced radiologist. Their results showed the difference in CAD output when used by a very experienced user and a less experienced one, with sensitivity being 88.6% and 70.5%, respectively and diagnostic accuracy 86% and 72%, respectively. The visual assessment by the experienced radiologist had higher specificity (96.4%) and diagnostic accuracy (91%) than all CAD approaches. Although this was the only study that assessed CAD performance based on the user’s thyroid ultrasound experience, these results suggest that thyroid ultrasound imaging experience may be a potential influencing factor of CAD diagnostic performance. More similar studies are warranted to validate this assertion.

Choi et al. [67] compared the diagnostic performance of the CAD system and an experienced radiologist for all nodules, nodules >1 cm, and the performance of the CAD system in nodule segmentation. The CAD system generally had a slightly higher sensitivity (90.7%) than radiologist assessment (88.4%) for all nodules but for nodules >1 cm, CAD sensitivity was 100% yet radiologist sensitivity was (92.9%). The specificity of CAD was, however, lower (71.8%) than that of the radiologist (97.4%). CAD assessment of nodules >1 cm had the least diagnostic accuracy (79.2%). Although these results suggest that CAD diagnostic performance may be dependent on the size of the nodules, it was the only study in this review that assessed the influence of size on grey scale CAD performance. Therefore, more studies with similar approaches would be helpful to validate these findings and ascertain the extent of the influence of nodule size on CAD performance.

Wang et al. [75] compared AI CAD based on a neural network and an experienced radiologist using ACR TIRADS. The AI CAD system had higher specificity (89.9%) than the radiologist (78%) whereas the sensitivity was comparable between both approaches, although that of the radiologist’s assessment was slightly higher (93.8%) than that of AI CAD (90.5%). The diagnostic accuracy of the CAD system was slightly higher (90.3%) than that of the radiologist (88.9%). These results concur with other studies that CAD has comparable diagnostic performance to that of radiologist assessment and furthermore has more potential for optimized sensitivity and specificity.

Gitto et al. [71] compared the diagnostic performance of an experienced radiologist and CAD system for the stratification of low to high suspicion thyroid nodules using the K-TIRADS [76]. CAD had a poor sensitivity of 21.4% while the specificity was 81.3%, whereas the radiologist visual assessment had higher sensitivity (78.6%) and lower specificity (66.7%). These findings contrasted other grey scale CAD studies in this review which generally showed that CAD had comparable or slightly higher sensitivity than radiologists’ visual assessment which had a higher specificity. The differences may be attributed to the difference in methodological approaches and potentially the choice of thyroid nodules in the latter study, which were mainly low to high suspicion nodules determined by FNAC.

#### 3.6.2. Performance Based on Different TIRADS Guidelines

Two studies evaluated the diagnostic performance of CAD and radiologists using different TIRADS. Gao et al. [70] compared the diagnostic performance of an AI CAD with that of radiologists using ATA, ACR, and KWAK TIRADS. Their results found a comparable sensitivity between CAD and radiologist assessments based on the three different TIRADS, however, the specificity of CAD was much lower (48.5%) than that of the radiologists (KWAK-75.7%; ATA-78.6%; and ACR-76.7%). The diagnostic accuracy of CAD was slightly lower (82.2%) than that of the radiologists using KWAK, ATA, and ACR TIRADS stratifications for subjective assessment which had comparable diagnostic accuracy (90.1%, 90.4% and 86%, respectively). The authors suggested that the AI CAD would be more helpful as a complementary tool in ruling out malignancy and excluding the need for FNAC, due to a high NPV (86.2%) and sensitivity (96.7%) despite the diagnostic accuracy and specificity being lower than that of the radiologists.

Reverter et al. [74] compared the diagnostic efficiency of a clinical expert using ATA TIRADS for grey scale ultrasound feature visual assessment and AmCAD CAD analysis based on three TIRADS guidelines within the system (ATA, EU and AACE/AME/ACE). The diagnostic performance of CAD differed based on the TIRADS used with CAD using ATA showing comparable sensitivity to the radiologist assessment (87%) whereas EU-TIRADS and AACE/ACE/AME-TIRADS yielded slightly lower sensitivity (85.2% and 81.5%, respectively). The visual assessment by the expert had much higher specificity (91.2%) than all the 3 TIRADS used in CAD assessment, with ATA-CAD yielding the better specificity (68.8%) amongst them. These findings concur with previously mentioned studies in this review which showed that CAD performance has comparable performance to radiologist visual assessment but has a lower specificity.

Limited evidence from these two studies suggests that the choice of TIRADS may potentially influence the diagnostic performance of CAD. However, because the studies used different approaches, one focusing on TIRADS-based visual assessment, while the other focused on TIRADS-based CAD assessment of thyroid nodules, future studies with similar methodological approaches are needed for CAD systems embedded with TIRADS so as to adequately assess the influence of the choice of TIRADS.

Overall, the highest sensitivity obtained for CAD approaches from studies included in this review was 100% for CAD of grey scale ultrasound features for nodules >1 cm, and 96.7% for all other nodules.

## 4. Discussion

### 4.1. Overview of Principal Findings

Human characterization of thyroid lesions relies on a qualitative assessment of ultrasound features based on established risk stratification guidelines. This approach is highly subjective and therefore prone to inter-observer variabilities even when the same risk stratification system is applied by different users. CAD approaches employ computational quantitative methods in image feature analysis thereby reducing the potential for human biases. In this study, we systematically reviewed the current literature on the diagnostic performance of ultrasound CAD approaches for thyroid nodule characterization in clinical settings. Studies included in this review focused on comparative analysis of grey scale CAD and visual assessment based on different criteria, Doppler ultrasound CAD, and CAD of sole ultrasound features such as echogenicity and calcification.

Based on the seven studies focused on grey scale ultrasound CAD, it was found that overall, CAD approaches generally perform comparably to qualitative assessments by radiologists in terms of sensitivity but have a lower specificity. These findings concur with those from a recent meta-analysis which evaluated the diagnostic performance of CAD and radiologists’ visual assessment of grey scale thyroid nodule ultrasound features [20]. However, unlike this current review, the meta-analysis only focused on grey scale features and did not include Doppler ultrasound studies. Limited evidence from the only study which assessed combined radiologist assessment of grey scale ultrasound features and CAD assessment suggests that a balanced sensitivity and specificity can be achieved with combined techniques than sole visual assessment or CAD assessment. This emphasizes the complementary role of CAD to the human assessment of ultrasound features, however, the lack of studies of combined techniques warrants more future studies in this area to sufficiently determine the diagnostic performance.

Overall, quantitative Doppler vascularity analysis approaches generally yielded balanced sensitivity and specificity than visual vascularity assessment which showed variable results in the three Doppler ultrasound CAD studies that determined the diagnostic performance of both approaches. Variable results for qualitative vascularity assessment can be attributed to the subjective grading of vascularity which is prone to interpreter bias. Quantitative vascularity analysis approaches based on segmentation of peripheral and central vascularity overcome limitations of subjective visual assessment of vascularity pattern and distribution [15,16,77]. Only one study demonstrated that the combination of Doppler ultrasound CAD with qualitative grey scale ultrasound features can result in an optimal sensitivity and specificity, but with reduced sensitivity compared to individual assessment of both qualitative and Doppler CAD [66]. The reduction in sensitivity may be attributed to the unknown influence of collinearity of different ultrasound features when they are assessed in combination. Although this is limited evidence on combined approaches, the findings concur with the only grey scale CAD study in this review which determined diagnostic performance of combined visual and CAD assessment. This suggests the potential for improved diagnostic performance with combined CAD techniques.

The diagnostic performance of CAD of sole features such as echogenicity and calcification was found to yield higher specificity than sensitivity with results showing features such as computed marked hypoechogenicity as highly specific. These findings concur with other previous grey scale thyroid ultrasound studies that indicated that marked hypoechogenicity has higher accuracy in the prediction for malignancy mostly when combined with other suspicious features [73,74]. However, due to the limited number of studies assessing CAD of these features in this review, generalizability may not be adequately achieved from these findings.

### 4.2. Potential Factors that May Influence CAD Diagnostic Performance

Limited evidence from this review suggests that the diagnostic performance of CAD may be influenced by the user’s ultrasound experience with more experienced radiologists yielding higher diagnostic performance in comparison to users with minimal or no thyroid ultrasound experience [68]. This can be attributed to ultrasound imaging being operator-dependent and highly dependent on the user’s technique and chosen settings on the ultrasound machine. CAD approaches involve automated or semi-automated region of interest (ROI) selection methods which may require the users to manually delineate the margins of the ROI. Furthermore, inexperience in the ultrasound technique may result in the acquisition of images of poor quality which may, in turn, result in wrong interpretation by the CAD system thereby resulting in misdiagnosis [64].

In this review, the diagnostic performance of CAD based on different TIRADS systems showed that ATA-TIRADS had the highest sensitivity. Although this evidence is based on two studies, these findings concur with some studies that indicated ATA- and KWAK-TIRADS have better diagnostic efficiency in comparison with other TIRADS systems in human visual assessment of thyroid nodules >1 cm [78,79,80]. Contrarily, some recent studies state that ACR-TIRADS has the lowest false-negative rate and is superior at reducing cases of unnecessary thyroid FNAC [81,82,83]. The differences in literature findings may be due to the differences in the criteria within the different TIRADS systems.

In the current review, two studies showed that the diagnostic performance of both grey scale and Doppler ultrasound CAD may potentially be influenced by the size of the nodule [67,69]. Doppler ultrasound CAD seems to perform more reliably than visual vascularity assessment for thyroid nodules <2 cm. This can be attributed to the highly subjective determination of intranodular vascularization by visual assessment. Conversely, grey scale ultrasound CAD appears to have higher sensitivity than specificity, especially for nodules >1 cm. Although a meta-analysis on the accuracy of thyroid ultrasound in malignancy determination suggested that thyroid nodule size is not a reliable predictor of malignancy [1], the results from this review, concur with recent findings that nodule size influences diagnostic performance of visual assessment, with higher sensitivity for nodules <2 cm, and higher specificity for nodules >2 cm by human assessment using ATA-TIRADS [84]. Similarly, a prior study indicated that for nodules <1 cm, the presence of three or more predictive features increases diagnostic accuracy while for nodules >1 cm a combination of two features can result in the diagnostic accuracy of about 90% [85]. However, due to the minimal evidence from this review, future studies evaluating the influence of nodule size on CAD performance could help validate these findings.

### 4.3. Clinical Implications and Suggested Directions for Future Research

CAD approaches may be helpful in preventing misdiagnosis when used as a second opinion particularly in thyroid cases with ultrasound features which may be ambiguous on visual assessment. CAD assessment of sole features, however, may have limited clinical value because features such as microcalcifications are highly specific to papillary thyroid cancer, thereby potentially excluding other thyroid cancers. CAD approaches of sole features may, however, have a potential role in determining the extent of features such as calcification thereby assisting in the sub-classification of thyroid nodules based on computed characteristic features. Because no single ultrasound feature is highly predictive of malignancy on its own, CAD of multiple ultrasound features is more diagnostically accurate and reliable.

Due to the high specificity of Doppler ultrasound CAD approaches, they have the potential for application in clinical practice in helping avoid unnecessary FNAC and surgery in otherwise benign thyroid nodules that are <2 cm and have increased intranodular vascularity. However, their routine clinical application may be limited by the lack of standardization in methodology and threshold determination. Therefore, the future development of Doppler ultrasound CAD should involve standardizing the approaches and embedding the software within the ultrasound system for real-time analysis for ease of clinical use and comparison of findings. Furthermore, as there are limited studies evaluating the diagnostic performance of quantitative thyroid vascularity determination techniques in combination with human assessment of grey scale ultrasound features, future studies with a focus on combined thyroid ultrasound CAD techniques, would aid in drawing objective inferences regarding the diagnostic performance of thyroid CAD approaches.

Because ultrasound imaging is an operator-dependent modality, the clinical application of thyroid ultrasound CAD in routine practice may require adequate training of CAD users in ultrasound image acquisition and CAD image selection and analysis in order to achieve optimal diagnostic performance. Furthermore, because varying TIRADS are incorporated within some CAD software, the choice of TIRADS may be limited by the software provider. Therefore, due to limited TIRADS choices in some CAD software, it may be beneficial to adopt a goal oriented TIRADS selection approach that best increases specificity or sensitivity or optimizes both for clinical practice. Additionally, for current CAD approaches, the choice of TIRADS for the CAD analysis may need to be made in consideration of the nodule size for optimized diagnostic accuracy, although this is an area for further research. As embedded TIRADS are structured for qualitative visual assessment thereby resulting in a bias for texture analysis in most CAD approaches, another research focus could be optimizing the TIRADS for CAD by developing diagnostic criteria for more quantitative features based on AI techniques.

### 4.4. Strengths and Limitations

The strength of this review was that a broad approach was adopted in evaluating different thyroid ultrasound CAD approaches. From the diverse studies included in this review, a general narrative overview of the diagnostic performance of ultrasound CAD for thyroid nodules and potential influencing factors could be identified from the findings from the different studies. To the best of our knowledge, there is currently limited literature on systematic reviews and narrative reviews on the diagnostic performance of different thyroid ultrasound CAD approaches and this review may be used to highlight potential areas for future studies.

The broad spectrum in the study design, study subjects and outcomes in the different selected studies for CAD of both grey scale and Doppler ultrasound features was also a limitation, in that it hindered a meta-analysis and group-based analysis. This can be attributed to the limited evidence of thyroid ultrasound CAD studies of similar approaches in the clinical setting. Although an extensive search strategy was used to ensure the screening of all relevant studies, some studies may have been missed particularly due to the English language restriction in our search criteria.

## 5. Conclusions

This review suggests that CAD of thyroid ultrasound features has a good diagnostic performance which is comparable to that of radiologists’ qualitative assessment with the potential for improved overall diagnostic accuracy when qualitative and quantitative approaches are combined. The nodule size, the experience of the operator and the choice of TIRADS system are potential influencers of CAD diagnostic performance. Future multi-center studies that compare similar CAD software based on standardized approaches and assess the diagnostic performance of combined Doppler ultrasound CAD and grey scale ultrasound CAD of the same thyroid nodules are recommended to further evaluate the clinical role of CAD in thyroid nodule characterization.

## Figures and Tables

**Table 1:** Main patient characteristics of the included studies.

Author(s)	Ref. Year	Patients Total (n)	Mean Age-Years (SD/Range)			Nodules (n)			Mean Size of Nodules-cm (SD)	
			Overall	Benign	Malignant	Total	Benign	Malignant	Benign	Malignant
Lyshchik et al.	2007 [69]	56	53.1 ± 11.6	NA	NA	86	40	46	NA	NA
Chen et al.	2011 [64]	225	NA	50.6 ± 12.52	46.7 ± 15.22	256	173	83	2.35 ± 0.98	1.94 ± 0.86
Wu et al.	2013 [62]	208	49.6 ± 13.4	51.0 ± 12.7	47.0 ± 14.2	238	159	79	NA	NA
Choi et al.	2015 [72]	85	52 (29–81)	NA	NA	99	21	78	NA	NA
Sultan et al.	2015 [73]	99	54 ± 15.5	56.6 ± 14.6	50.7 ± 16.4	100	58	42	1.81 ± 0.73	1.77 ± 0.74
Wu et al.	2016 [63]	333	48.37 (11–81)	NA	NA	411	254	157	NA	NA
Baig et al.	2017 [66]	111	NA	51.2 ± 12	56.6 ± 17.6	111	84	27	NA	NA
Gao et al.	2018 [70]	262	NA	48.4 ± 12.3	43.2 ± 10.4	342	103	239	1.7 ± 1.4	1 ± 0.7
Choi et al.	2017 [67]	89	45.3	NA	NA	102	59	43	1.5 ± 0.8	0.9 ± 0.4
Gitto et al.	2019 [71]	62	60 ± 12	NA	NA	62	48	14	NA	NA
Yoo et al.	2018 [65]	50	43.2 (22–81)	NA	NA	117	67	50	1.2 ± 1.0	1.1 ± 0.8
Jeong et al.	2019 [68]	76	46	NA	NA	100	56	44	1.8 ± 0.8	1.5 ± 0.8
Reverter et al.	2019 [74]	300	NA	55 ± 11	56 ± 12	300	165	135	2.8 ± 0.4	3.2 ± 1.0
Wang et al.	2019 [75]	276	46.3 (20–71)	50 ± 10.6	44.3 ± 11.5	351	109	242	3.37 ± 1.81	1.17 ± 0.87

NA-not available.

**Table 2:** Main features of the diagnostic tools used in the included studies.

Author(s)	Type of Ultrasound Machine	Type of CAD	Reference Standard	Diagnosis Parameter
Lyshchik et al., 2007 [69]	Siemens Sonoline Elegra with a 5–9MHz linear array transducer (7.5L40)	Algorithm for manual segmentation of tumor and Doppler quantification in MATLAB	Histopathology	Doppler–visual and quantitative intranodular vascularization (vascular index-VI)
Chen et al., 2011 [64]	Philips HDI 5000 (2000 model) with a 5–12 MHz linear probe (L12–5)	AmCAD-UT (grey scale CAD of microcalcifications)	FNAC (75)/Histopathology (181)	Qualitative and computed calcification analysis (calcification index-CI)
Wu et al., 2013 [62]	Philips HDI 5000 (2000 model) with a 5–12 MHz linear probe (L12–5)	Stand-alone AmCAD-UV (Doppler CAD)	FNAC/Histopathology	Doppler–quantitative intranodular vascularization (vascular index-VI)
Choi et al., 2015 [72]	Philips HDI 5000	CAD based on artificial intelligence for calcification assessment	Histopathology	Computed grey scale calcification analysis
Sultan et al., 2015 [73]	Philips HDI 5000 (68), Philips iu22 (30), GE LOGIC E9, GE LOGIC 9	IDL-based software computer program for vascular analysis	Histopathology/FNAC	Qualitative and quantitative vascular area analysis
Wu et al., 2016 [63]	Philips HDI 5000 (2000 model) with a 5–12 MHz linear probe (L12–5)	Stand-alone AmCAD-UT (grey scale CAD of echogenicity)	FNAC/Histopathology	Qualitative and quantitative echogenicity analysis (echogenicity index-EI)
Baig et al., 2017 [66]	Supersonic Imagine Aixplorer with 4–15 MHz linear transducer	Custom-developed Doppler algorithm for use in MATLAB	FNAC (62–benign)/Histopathology (49)	Quantitative regional Doppler vascularization analysis (vascular index-VI)
Gao et al., 2018 [70]	Philips HDI 5000, GE Logiq 9 and GE Logiq 7 with a 5–12 MHz or 8–15 MHz linear array transducer	CAD-based on artificial intelligence	Histopathology	Qualitative and computed grey scale analysis
Choi et al., 2017 [67]	Samsung RS80A with 3–12 MHz linear transducer	S-Detect for Thyroid CAD embedded in Samsung US scanner	Histopathology/FNAC/US findings	Qualitative and computed grey scale feature analysis
Gitto et al., 2019 [71]	Samsung RS80A with 3–8 MHz linear transducer	S-Detect for Thyroid CAD embedded in Samsung US scanner	FNAC	Qualitative and computed grey scale feature analysis
Yoo et al., 2018 [65]	Samsung RS80A with a 5–12 MHz linear probe (Samsung Medison Co., Ltd.)	S-Detect for Thyroid CAD embedded in Samsung US scanner	FNAC (14)/Histopathology (103)	Qualitative and computed grey scale feature analysis
Jeong et al., 2019 [68]	Samsung RS80A with 5–12 MHz linear transducer	S-Detect for Thyroid CAD embedded in Samsung US scanner	Histopathology/FNAC	Qualitative and computed grey scale feature analysis
Reverter et al., 2019 [74]	GE Logiq E9 with 5–15 MHz linear transducer	AmCAD-UT	Histopathology	Qualitative and computed grey scale analysis
Wang et al., 2019 [75]	GE Logiq E8, Philips iE Elite, and Philips iU22 with a 6–15 MHz, 3–11 MHz or 5–12 MHz linear array transducer	CAD-based on artificial intelligence	Histopathology	Qualitative and computed grey scale analysis

CAD: computer-aided diagnosis, IDL: Interactive data language, US-ultrasound; FNAC: Fine-needle aspiration cytology.

**Table 3:** Diagnostic performance of thyroid ultrasound computer-aided diagnosis (CAD) for characterization of malignant and benign thyroid nodules.

Author(s)	Diagnostic Criteria	SEN (%)–95% CI	SPEC (%)–95% CI	PPV (%)–95% CI	NPV (%)–95% CI	DA (%)–95% CI	AUC–95 CI
Lyshchik et al. [69]	Visual vascularization	65.2 (49.75–78.65)	52.5 (36.13–68.49)	* 61.22 (51.71–69.95)	* 56.76 (44.48–68.25)	58.9 (48.17–69.78)	ND
	Visual <2 cm	65.5 (45.67–82.06)	85.7 (57.19–98.22)	* 90.48 (71.94–97.24)	* 54.55 (41.02–67.43)	72.1 (56.33–84.67)	
	Normalized VI >0.14 in <2 cm	72.4 (52.76–87.27)	100 (76.84–100)	* 100	* 63.64 (49.25–75.94)	86.2 (66.60–91.61)	
	Weighted VI >0.24 in <2 cm	69 (49.17–84.72)	100 (76.84–100)	* 100	* 60.87 (47.48–72.80)	84.5 (63.96–89.96)	
Chen et al. [64]	Qualitative calcification	48.2 (37.08–59.44)	89 (83.38–93.26)	67.8 (56.59–77.27)	78.2 (74.30–81.60)	75.8 (70.06–80.90)	ND
	CI threshold at 0.0089	63.9 (51.69–73.86)	80.9 (71.35–87.59)	* 71.43 (61.99–79.31)	* 73.87 (67.58–79.32)	* 72.93 (65.84–79.25)	0.763
	CI threshold at 0.00488	80 (69.20–87.96)	55 (44.74–64.78)	* 57.80 (51.83–63.55)	* 77.78 (68.59–84.87)	* 65.75 (58.34–72.63)	0.763
Wu et al., 2013 [62]	Mean VI at 37.056 threshold	84.8 (74.97–91.90)	40.9 (33.16–48.95)	41.6 (37.80–45.53)	84.4 (75.69–90.40)	55.5 (48.90–61.88)	0.711
	Mean VI at 10.330 threshold	45.6 (34.31–57.17)	83.7 (76.97–89.03)	58.06 (47.48–67.95)	75.6 (71.42–79.29)	71 (64.80–76.69)	0.711
	Central VI at 32.285 threshold	83.5 (73.51–90.94)	41.5 (33.76–49.58)	41.5 (37.60–45.53)	83.5 (74.93–89.61)	55.5 (48.90–61.88)	0.71
	Central VI at 5.453 threshold	40.5 (29.60–52.15)	89.3 (83.43–93.65)	65.3 (52.74–76.05)	75.1 (71.42–78.51)	73.1 (67.00–78.63)	0.71
	Overall VI at 42.014 threshold	78.5 (67.80–86.94)	40.3 (32.56–48.31)	39.2 (35.46–43.67)	78.8 (70.35–85.66)	52.9 (46.39–59.42)	0.693
	Overall VI at 15.755 threshold	40.5 (29.60–52.15)	83 (76.26–88.50)	53.3 (43.40–64.69)	73.6 (69.80–77.34)	68.9 (62.61–74.73)	0.693
Choi et al., 2015 [72]	0.64 threshold	83 (73.19–90.82)	82.4 (58.09–94.55)	* 94.2 (87.00–97.53)	* 56.7 (43.30–69.13)	82.8 (73.94–89.67)	0.83
Sultan et al. [73]	Qualitative vascularity	67.5 (50.45–80.43)	88.1 (76.70–95.01)	* 80 (65.91–89.22)	* 78.5 (70.15–84.95)	* 79 (69.71–86.51)	ND
	Central vascular fraction area	90 (77.38–97.34)	88 (76.70–95.01)	84 (72.91–91.63)	92 (83.32–97.02)	89 (81.17–94.38)	
	Central flow volume index	50 (34.19–65.81)	62 (48.37–74.49)	48 (37.91–59.88)	63 (54.38–71.14)	56 (46.71–66.86)	
Wu et al., 2016 [63]	Visual hypoechogenicity	89.8 (83.98–94.06)	31.9 (26.20–38.01)	44.9 (42.46–47.37)	83.5 (75.47–89.28)	54 (49.06–58.91)	ND
	Comp. hypoechogenicity (EI_N–T_)	79.6 (72.46–85.62)	52.4 (46.03–58.64)	50.8 (47.03–54.58)	80.6 (74.91–85.26)	62.8 (57.90–67.46)	0.7
	Mark. hypoechogenicity (EI_N–M_)	33.1 (25.82–41.07)	93.3 (89.50–96.05)	75.4 (64.75–83.59)	69.3 (66.80–71.69)	70.3 (65.64–74.69)	0.77
Baig et al. [66]	Visual grey scale evaluation	96.3 (81.03–99.91)	46.4 (35.47–57.65)	36.6 (31.84–41.67)	97.5 (84.90–99.63)	58.6 (48.82–67.83)	ND
	Combined VI at 22% off-set	70.4 (49.82–86.25	71.4 (60.53–80.76)	44.2 (34.28–54.58	88.2 (80.50–93.16)	71.2 (61.80–79.37)	ND
	Combined VI + visual GSU	66.7 (46.04–83.48)	83.3 (73.62–90.58)	56.3 (42.65–68.97)	88.6 (81.90–93.04)	79.3 (70.55–86.39)	ND
Gao et al. [70]	CAD	96.7 (93.51–98.54)	48.5 (38.58–58.60)	81.3 (78.30–84.04)	86.2 (75.45–92.71)	82.2 (77.69–86.07)	0.73
	Radiologist–KWAK	96.2 (92.97–98.26)	75.7 (66.29–83.64)	90.2 (86.73–92.83)	89.7 (81.90–94.32)	90.1 (86.39–93.02)	0.87
	Radiologist–ATA	95.4 (91.91–97.68	78.6 (69.47–86.10)	91.2 (87.73–93.76)	88 (80.39–92.97)	90.4 (86.72–93.26)	0.83
	Radiologist–ACR	90.0 (85.43–93.46)	76.7 (67.34–84.46)	90.0 (86.29–92.73)	76.7 (68.94–83.00)	86 (81.83–89.47)	0.86
Choi et al., 2017 [67]	CAD–all nodules	90.7 (77.9–97.4)	74.6 (61.6–85.0)	72.2 (58.4–83.5)	91.7 (80.0–97.7)	81.4	0.83 (0.74–0.89)
	Radiologist–all nodules	88.4 (74.9–96.1)	94.9 (85.9–98.9)	92.7 (80.1–98.5)	91.8 (81.9–97.3)	92.2	0.92 (0.84–0.96)
	CAD >1 cm nodules	100 (76.8–100.00)	71.8 (55.1–85.0)	56 (34.9–75.6)	100 (87.7–100)	79.2	0.86 (0.74–0.94)
	Radiologist >1 cm nodules	92.9 (66.1–99.8)	97.4 (86.5–99.9)	92.9 (66.1–99.8)	97.4 (86.5–99.9)	96.2	0.95 (0.85–0.99)
Gitto et al. [71]	CAD	21.4 (4.7–50.8)	81.3 (67.4–91.1)	25 (9.4–51.6)	78 (72.3–82.8)	67.7	ND
	Radiologist-K-TIRADS	78.6 (49.2–95.3)	66.7 (51.6–79.6)	40.7 (29.8–52.8)	91.4 (79.3–96.7)	69.4	ND
Yoo et al. [65]	CAD	80 (66.28–89.97)	88.1 (77.82–94.70)	83.3 (72.00–90.67)	85.5 (77.09–91.18)	84.6 (76.78–90.62)	0.84 (0.76–0.90)
	Radiologist	84 (70.89–92.83)	95.5 (87.47–99.07)	93.3 (82.15–97.71)	88.9 (80.88–93.80)	90.6 (83.80–95.21)	0.90 (0.83–0.95)
	Radiologist + CAD	92 (80.77–97.78)	85.1 (74.26–92.60)	82.1 (72.08–89.12)	93.4 (84.70–97.35)	88 (80.74–93.30)	0.89 (0.81–0.94)
Jeong et al. [68]	Expert Radiologist	84.1 (69.93–93.36)	96.4 (87.69–99.56)	94.9 (82.50–98.64)	88.5 (79.61–93.84)	91 (83.60–95.80)	ND
	Expert Radiologist using CAD	88.6 (75.44–96.21)	83.9 (71.67–92.38)	81.3 (70.24–88.84)	90.4 (80.34–95.58)	86 (77.63–92.13)	0.863
	User 1 using CAD	70.5 (54.80–83.24)	80.4 (67.57–89.77)	73.8 (61.61–83.19)	77.6 (68.30–84.76)	76 (66.43–83.98)	0.754
	User 2 using CAD	75 (59.66–86.81)	73.2 (59.70–84.17)	68.8 (58.01–77.80)	78.8 (68.57–86.43)	74 (64.27–82.26)	0.741
	User 3 using CAD	70.5 (54.80–83.24)	73.2 (59.70–84.17)	67.4 (56.28–76.84)	75 (66.05–83.64)	72 (62.13–80.52)	0.718
Reverter et al. [74]	Expert–ATA	87 (79.75–91.90) *	91.2 (85.4–94.82) *	90.5 (82.74–92.70) *	90.9 (84.39–92.78) *	* 89.00 (84.90–92.31)	0.88
	CAD–ATA	87 (79.75–91.90) *	68.8 (61.44–76.04) *	64.5 (64.40–74.42) *	86.3 (80.28–90.79) *	* 77.00 (71.82–81.64)	0.72
	CAD–EU	85.2 (78.05–90.71) *	50.2 (42.43–58.17) *	50.1 (54.22–62.41) *	82.6 (72.93–86.47) *	* 66.00 (60.33–71.35)	0.71
	CAD–AACE/AME/ACE	81.5 (73.89–87.64)	53.2 (45.42–61.13) *	51.8 (54.36–63.15) *	80.8 (70.62–83.75) *	* 66.00 (60.33–71.35)	0.7
Wang et al. [75]	CAD	90.5 (86.08–93.88)	89.9 (82.66–94.85)	95.2 (91.90–97.22)	81 (74.19–86.33)	90.3 (86.73–93.20)	0.902 (0.866–0.931)
	Radiologist	93.8 (89.98–96.49)	78 (69.03–85.35)	90.4 (86.90–93.10)	85 (77.46–90.33)	88.9 (85.12–91.98)	0.859 (0.818–0.894)

* calculated value based on available data from the study-may not correspond exactly to the given value. Abbreviations: SEN, sensitivity; SPEC, specificity; PPV, positive predictive value; NPV, negative predictive value; DA, diagnostic accuracy; AUC, area under curve; CI, confidence interval; ND, not determined; GSU, grey scale ultrasound; comp, computed; mark., marked. EI_N-T_-Echogenicity index (nodule–thyroid tissue); EI_N-M_-Echogenicity index (nodule-muscle), K-TIRADS-Korean TIRADSATA: American Thyroid Association EU: European Thyroid Association; AME: Associazione Medici Endocrinologi; ACE: American College of Endocrinology.

**Table 4:** Quality assessment of diagnostic accuracy studies (QUADAS) bias results.

Author (S)	Patient Selection	Index Test	Reference Standard	Flow and Timing
Lyshchik et al., 2007 [69]	High	Low	Low	Low
Chen et al., 2011 [64]	Low	Low	Low	Low
Wu et al., 2013 [62]	Low	Low	Low	Low
Choi et al., 2015 [72]	High	High	Low	Low
Sultan et al., 2015 [73]	High	Low	Low	Unclear
Wu et al., 2016 [63]	Unclear	Low	Low	Low
Baig et al., 2017 [66]	Low	Low	Low	Low
Gao et al., 2018 [70]	High	Low	High	Low
Choi et al., 2017 [67]	Low	Low	Low	Low
Gitto et al., 2019 [71]	Low	Low	Low	Low
Yoo et al., 2018 [65]	Low	Low	Low	Low
Jeong et al., 2019 [68]	Low	Low	Low	Low
Reverter et al., 2019 [74]	High	Low	Low	Low
Wang et al., 2019 [75]	High	Unclear	Low	Low

**Table 5:** QUADAS Applicability Results.

Author (S)	Patient Selection	Index Test	Reference Standard
Lyshchik et al., 2007 [69]	Low	Low	Low
Chen et al., 2011 [64]	Low	Low	Low
Wu et al., 2013 [62]	Low	Low	Low
Choi et al., 2015 [72]	High	Unclear	Low
Sultan et al., 2015 [73]	Low	Low	Low
Wu et al., 2016 [63]	Low	Low	Low
Baig et al., 2017 [66]	Low	Low	Low
Gao et al., 2018 [70]	Low	Low	High
Choi et al., 2017 [67]	Low	Low	Low
Gitto et al., 2019 [71]	Low	Low	Low
Yoo et al., 2018 [65]	Low	Low	Low
Jeong et al., 2019 [68]	Low	Low	Low
Reverter et al., 2019 [74]	Low	Low	Low
Wang et al., 2019 [75]	Low	Low	Low

## Supplementary Materials

The following are available online at https://www.mdpi.com/2072-6694/11/11/1759/s1, Figure S1: QUADAS Risk of bias summary, Figure S2: QUADAS Applicability Concerns. Table S1: PRISMA 2009 checklist.
